# Factors associated with acute kidney injury in the Helsinki Burn Centre in 2006–2015

**DOI:** 10.1186/s13049-018-0573-3

**Published:** 2018-12-13

**Authors:** I. Rakkolainen, J. V. Lindbohm, J. Vuola

**Affiliations:** 1 Helsinki Burn Centre, Department of Plastic Surgery, Helsinki University Hospital and University of Helsinki, PO. Box 800, FI-00029 HUS Helsinki, Finland; 2 Department of Public Health, University of Helsinki, Helsinki, Finland

**Keywords:** Burn injury, Acute kidney injury, Renal replacement therapy

## Abstract

**Background:**

Acute kidney injury (AKI) is a common complication in severe burns and can lead to significantly poorer outcomes. Although the prognosis has improved in recent decades, the mortality of AKI remains considerable. We investigated the factors that increase the risk of AKI and death after severe burn injury.

**Methods:**

Intensive care patients with ≥20% burned total body surface area (TBSA%) between January 2006 and December 2015 treated in Helsinki Burn Centre were enrolled retrospectively. Patients who arrived > 36 h after burn injury or died < 48 h from arrival were excluded. A total of 187 patients were included in the final analysis. Serum creatinine ≥120 μmol/l (1.4 mg/dl) was the criterion for AKI.

**Results:**

Fifty-one patients (27.3%) developed AKI during hospital stay and 21 (11.2%) required renal replacement therapy (RRT); 37 patients (19.8%) died during hospital stay. Mortality was significantly higher in the AKI group (52.9%) than in the AKI-negative group (7.4%). The Abbreviated Burn Severity Index (ABSI), Baux, and the modified Baux score were nearly equivalent in predicting mortality during ICU stay (AUC: 0.83–0.84). The risk of death and AKI were minimal with Baux scores < 80. LD_50_ was 112 for Baux score in all patients. In flame burns, the risk of death increased rapidly after Baux score > 80. Multivariate logistic regression model detected age, TBSA%, sepsis, and rhabdomyolysis as independent risk factors for AKI. Age (per 10 yrs. OR 1.99), TBSA% (per 10% OR 1.64), and AKI predicted mortality during hospital stay; AKI had an odds ratio of (OR) of 5.97 (95% confidence interval [CI] 2.2–16.2).

**Conclusions:**

Age, TBSA%, and AKI were the strongest independent factors in predicting outcome in severe burns. Even a major burn (> 50% TBSA) has a relatively good prognosis without simultaneous AKI. Prognosis is poorer even in minor burns for patients with AKI.

**Electronic supplementary material:**

The online version of this article (10.1186/s13049-018-0573-3) contains supplementary material, which is available to authorized users.

## Background

Acute kidney injury (AKI) is a common complication of severe burns and has a high mortality rate. Data collected from studies published in 2007–2016 indicates an AKI incidence in severe burns of approximately 40% [1]. Since the early 1960s, the prognosis of patients with severe burns and AKI has improved significantly [2] although mortality remains high (up to 45%) [3–5]. The known risk factors for AKI are increased age, large burned TBSA%, flame burn, thickness of burn, inhalation injury, and sepsis [1]. Early-onset and late-onset AKI have different aetiologic factors: Early AKI is mainly associated with circulation deficit and cardiac dysfunction causing insufficient perfusion to kidneys, whereas late AKI is often caused by sepsis and multiple organ failure (MOF) [6, 7], their conflicting results on their importance in mortality have been published [8–13]. The aim of this study was to investigate which factors are related to AKI and mortality after severe burn injury, the mortality between early and late AKI and if renal replacement therapy (RRT) improves the prognosis of patients with poor kidney function. We also wanted to establish whether the prognosis of AKI patients has changed since the previous publication from our institution from 1988 to 2001 [3].

## Materials and methods

**Fig. 1:**
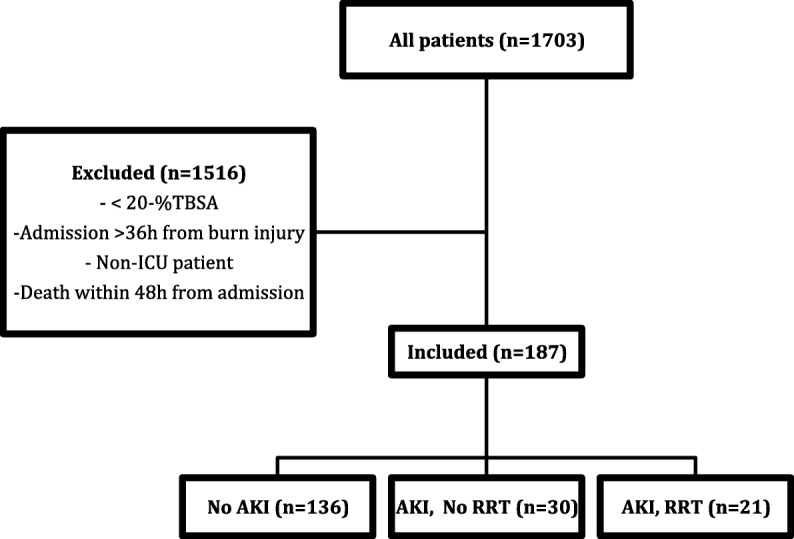
Overview of patients. TBSA, total body surface area; ICU, intensive care unit; AKI, acute kidney injury; RRT, renal replacement therapy

We retrospectively identified all intensive care patients admitted to Helsinki Burn Centre with ≥20% TBSA between January 2006 and December 2015. Patients who died < 48 h after admission or who arrived at the burn unit more than > 36 h after injury were excluded. Study groups and a flowchart of patient selection are shown in Fig. 1. The following parameters were collected during ICU stay: age, sex, burn mechanism, TBSA%, time in ICU, pre-existing comorbidity (chronic cardiac-, pulmonary-, hepatic-, renal- or neurological illness, excluding arterial hypertension without complications), sepsis, inhalation injury, patient intubated on arrival, need for escharotomies or fasciotomies, ABSI score [14], Baux score [15], the modified Baux score [16], presence of AKI or rhabdomyolysis, and need for RRT. Baux and ABSI scores were determined upon arrival. Patient considered having sepsis, if it was mentioned in patient’s medical records.

Serum creatinine (SCr) ≥120 μmol/l (1.4 mg/dl) was the criterion for AKI. We did not include patients as AKI patients if their SCr normalized < 120 μmol/l within 48 h. These patients were considered to have SCr increase due to dehydration. SCr was tested daily during study. AKI was considered ‘early’ when encountered within 5 days post burn and ‘late’ after 5 days [6]. Rhabdomyolysis was considered positive when plasma creatinine kinase (P-CK) was ≥5000 IU [17].

Patients received the following standard burn care: resuscitation by Parkland formula (4 ml x kg x TBSA%, no colloids within first 8 h), urine output target 0.5 ml/kg/hour, preventive escharotomies or fasciotomies, and administration of vasoactive agents when necessary. Bronchoscopy was performed at early stage when burn mechanism included a possibility of inhalation injury. The general guidelines for consideration of RRT initiation were the following: hyperkalaemia (plasma potassium > 6.5 mmol/l), severe acidosis (arterial pH < 7.15), diuresis < 200 ml/12 h, fluid retention with anuria including extremity swelling, severe pulmonary oedema, increased intra-abdominal pressure, plasma urea > 35 mmol/l or SCr > 500 μmol/l (5.7 mg/dl). These criteria were not absolute; a combination of the different parameters and the patient’s general condition were all considered individually. RRT was not given in certain cases, although the patient met the criteria for initiation, if the overall prognosis was deemed poor.

Data were analysed with IBM SPSS Statistics for Macintosh, Version 22.0 (Armonk, NY, USA, IBM Corp.) and STATA, Version 12.0 (Stata Corp. LP College Station, TX, USA). Student’s t-test or Mann-Whitney U-test was used for continuous variables and Chi-square test or Fischer’s exact test for dichotomous variables. Logistic regression estimated odds ratios (ORs) for AKI and death in multivariate adjusted models. Based on literature, the fully adjusted model included age, TBSA%, sex, inhalation injury, and comorbidities. Due to differing disease aetiologies, multivariate analysis was performed separately for non-flame and flame burns. Based on multivariate logistic regression models, we predicted the probability of AKI and death against the Baux score for different burn types. The area under the curve (AUC) for scoring formulas was determined by the receiver operating characteristic (ROC) method. A *P*-value less than 0.05 (*P* < 0.05) was considered a statistically significant difference. In unadjusted models, the differences between studied outcomes were searched in explorative fashion. The study received research permit from Helsinki University Hospital.

## Results

A total of 1703 patients were treated in Helsinki Burn Centre between January 2006 and December 2015. Of these, 187 patients, 11% fulfilled the inclusion criteria. Fifty-one patients (27.3%) had AKI during hospital stay and 21 patients (11.2%) required RRT. Thirty-seven patients (19.8%) died during hospital stay. The median age of all patients was 48 years and median TBSA% was 33%. Demographic data are presented in Table 1.

**Table 1:**
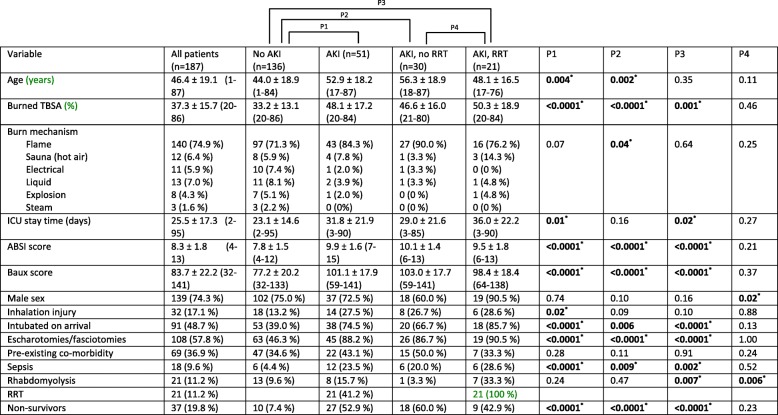
Demographic data of study groups

### Comparison of patients with and without AKI

None of the AKI patients had pre-existing chronic kidney disease according to available medical records. Patients with AKI were significantly older and had significantly more severe burn injury, longer ICU stay, higher ABSI and Baux scores, higher proportion of inhalation injury, escharotomies or fasciotomies, sepsis, need for intubation, and greater mortality than patients without AKI. Twenty-one (41.2% of AKI patients) received RRT during hospital stay. Flame burns were more common in the AKI group not receiving RRT than in the group without AKI (Table 1).

Thirty-four patients (66.7% of AKI patients) had early AKI. All AKIs caused by hot air in sauna and 88% of those induced by rhabdomyolysis were classified as early. Four out of 12 hot-air sauna patients suffered from AKI, three of them required RRT; all patients survived. Seventeen patients (33.3% of AKI patients) had late AKI. Late AKI patients had a significantly higher mean ABSI score (10.6 vs. 9.5) and TBSA% (57% vs. 44%) compared with the early AKI group (Additional file 1: Table S1). Age, TBSA%, sepsis, and rhabdomyolysis were independent risk factors for AKI in multivariate logistic regression model (Table 2).

**Table 2:** Risk factors of AKI and death from multivariate models

	OR (95%CI) for AKI	OR (95%CI) for death	OR (95%CI) for death with AKI	OR (95%CI) for death in AKI patients
Age (per 10y increase)	1.80 (1.37–2.37)	1.91 (1.40–2.61)	1.64 (1.18–2.27)	1.30 (0.99–1.90)
TBSA (per 10% increase)	2.16 (1.61–2.88)	2.36 (1.71–3.26)	1.99 (1.41–2.80)	1.49 (0.98–2.27)
Comorbidities	0.88 (0.37–2.09)	0.95 (0.38–2.39)	1.03 (0.39–2.74)	NA
Inhalation injury	2.46 (0.94–6.40)	1.77 (0.66–5.00)	1.33 (0.43–4.06)	NA
Sepsis	6.69 (1.71–26.26)	1.03 (0.26–4.09)	0.51 (0.12–2.19)	NA
Rhabdomyolysis	3.94 (1.10–14.06)	2.55 (0.66–9.83)	1.83 (0.43–7.72)	NA
AKI	NA	NA	5.97 (2.20–16.20)	NA

*AKI* acute kidney injury, *NA* not available, *TBSA* total body surface area

### Use of RRT in AKI patients

A total of 21 patients (11.2% of all patients and 41.2% of AKI patients) required RRT during hospital stay. Continuous renal replacement therapy (CRRT) was used for eight (38.1%), intermittent haemodialysis (IHD) for nine (42.9%), and CRRT following IHD for four (19%) of RRT patients. 90.5% of RRT patients were men. Rhabdomyolysis required significantly more often RRT when compared with the remainder of the AKI group. There were no significant differences in age, mortality, or severity of injury between AKI groups (ABSI, Baux score, TBSA%) (P4 in Table 1). 18 patients (86% of RRT patients) had early AKI. A decision to withhold RRT was made for 11 patients (37% of AKI patients that did not receive RRT); seven (64%) of these patients died. 78% of non-survivors in the AKI without RRT group (14 patients) had a decision to withhold RRT in all circumstances, or their condition was too poor to initiate RRT.

### Survivors vs. non-survivors

A total of 37 patients (19.8% of all patients) died during hospital stay. Flame burn occurred more frequently in non-survivors than in survivors (Table 3). Among all AKI patients, age, TBSA%, ABSI, or Baux score did not differ significantly between survivors and non-survivors. In RRT patients, the mean Baux score was significantly higher in non-survivors than in survivors (108 vs. 91). 50% of survivors in AKI group (12 patients) received RRT compared with 33% in non-survivors (nine patients). Advanced age, TBSA%, and AKI were risk factors for death in multivariable logistic regression model. AKI increased the risk of death with an OR of 5.97 (Table 2).

**Table 3:** Demographic data of non-survivors vs. survivors

Variable	Survivors (*n* = 150)	Non-survivors (*n* = 37)	*P*
Age (years)	44.3 ± 19.0 (1–84)	54.9 ± 17.4 (14–87)	**0.002** ^*^
Burned TBSA (%)	34.2 ± 13.4 (20–84)	49.6 ± 18.5 (20–86)	**< 0.0001** ^*^
Burn mechanism			
Flame	107 (71.3%)	33 (89.2%)	**0.03** ^*^
Sauna (hot air)	11 (7.3%)	1 (2.7%)	
Electrical	11 (7.3%)	0 (0%)	
Liquid	10 (6.7%)	3 (8.1%)	
Explosion	8 (5.3%)	0 (0%)	
Steam	3 (2.0%)	0 (0%)	
ICU stay time (days)	27.5 ± 17.2 (2–95)	17.5 ± 15.5 (3–51)	**0.002** ^*^
ABSI score	7.9 ± 1.6 (4–13)	10.1 ± 1.5 (6–13)	**< 0.0001** ^*^
Baux score	78.6 ± 20.5 (32–141)	104.6 ± 16.0 (59–138)	**< 0.0001** ^*^
Male sex	113 (75.3%)	26 (70.3%)	0.53
Inhalation injury	22 (14.7%)	10 (27.0%)	0.09
Intubated on arrival	69 (46.0%)	22 (59.5%)	0.20
Escharotomies/fasciotomies	78 (52.0%)	30 (81.1%)	**0.001** ^*^
Pre-existing co-morbidity	52 (34.7%)	17 (45.9%)	0.20
Sepsis	12 (8.0%)	6 (16.2%)	0.21
Rhabdomyolysis	16 (10.7%)	5 (13.5%)	0.57
AKI	24 (16.0%)	27 (73.0%)	**< 0.0001** ^*^
RRT	12 (8.0%)	9 (24.3%)	**0.009** ^*^

*) Statistically significant difference, *p* < 0.05

Data are reported as mean ± SD, (interval) or percentage, when appropriate. *ABSI* Abbreviated Burn Severity Index, *AKI* acute kidney injury, *ICU* intensive care unit, *RRT* renal replacement therapy, *TBSA* total body surface area

**Fig. 2:**
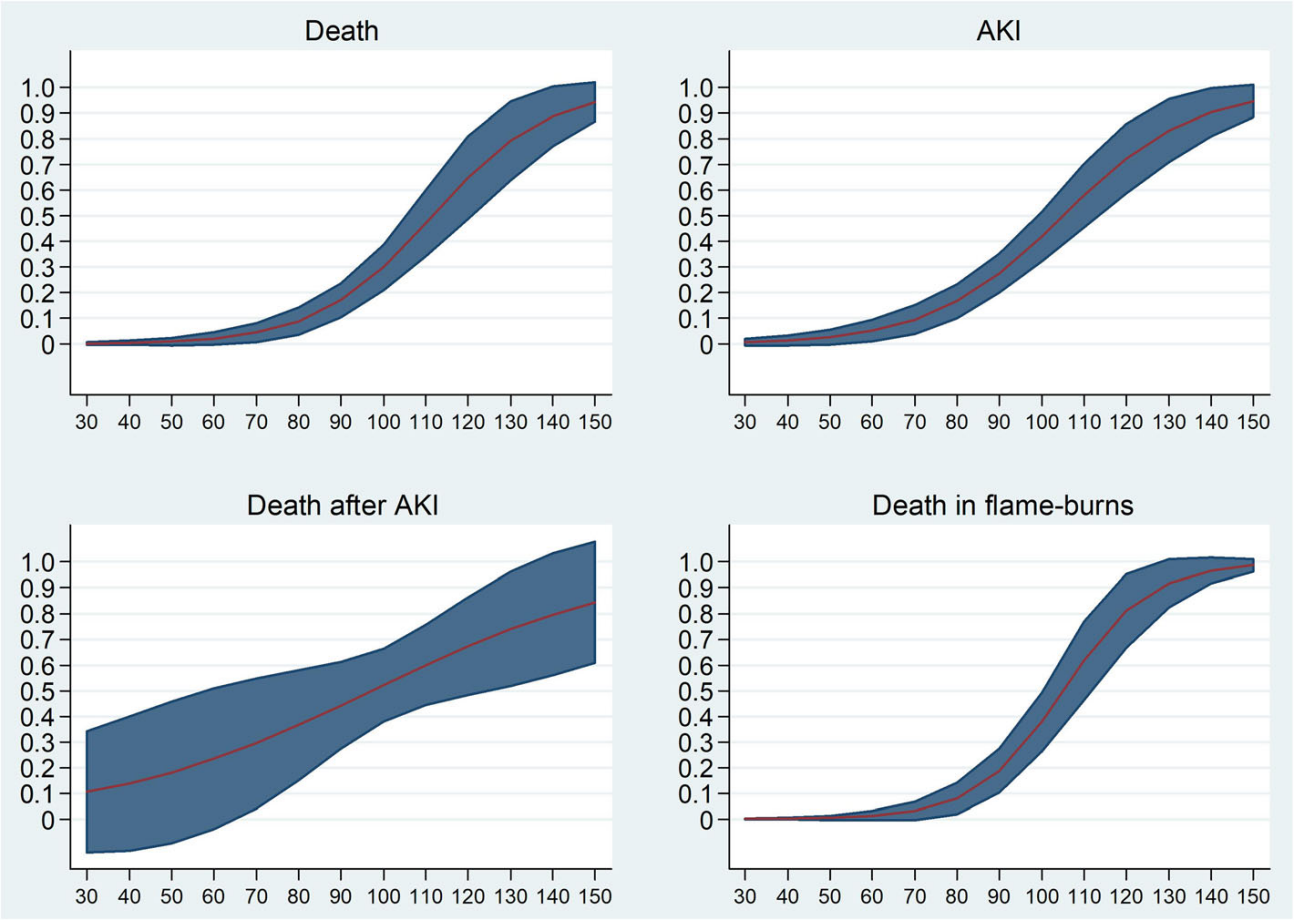
Baux score in x-axis and probability in y-axis. Death and AKI in all ICU patients (upper row); probability of death in AKI patients and –in patients with flame burn (lower row). Baux score predicting 50% chance for each endpoint (AKI, death) is marked with vertical line. Marked area around the curve shows 95% confidence intervals. AKI, acute kidney injury; ICU, intensive care unit

**Fig. 3:**
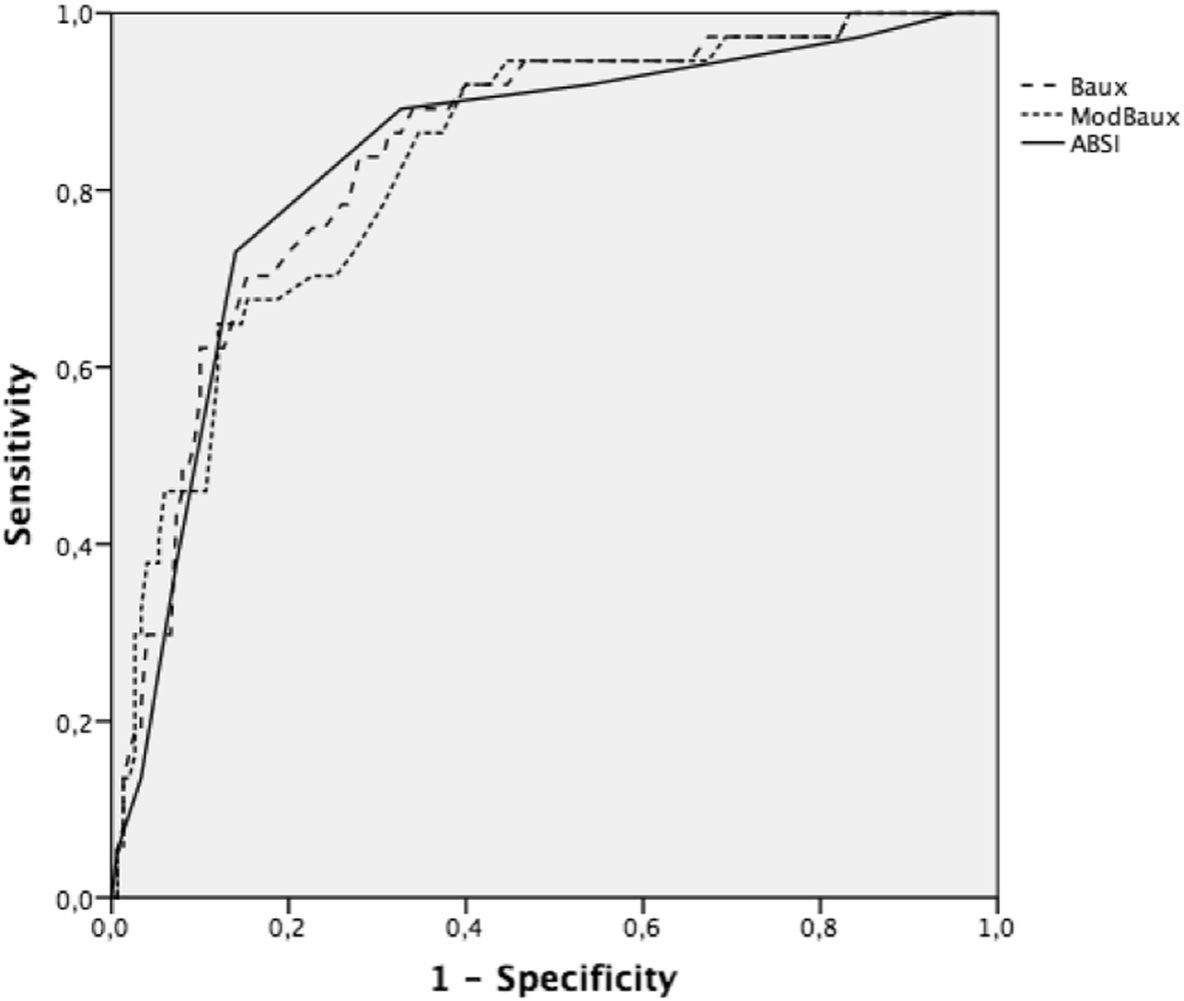
ROC-curves for ABSI-, Baux- and modified Baux scores predicting death during hospital stay. ABSI, Abbreviated Burn Severity Index

The LD_50_ for Baux score was 112 in all ICU patients (Fig. 2). A receiver operating characteristic (ROC) curve in predicting death during ICU stay indicated 0.84 (95% CI 0.78–0.91) for Baux score, 0.83 (95% CI 0.76–0.90) for modified Baux score, and 0.83 (95% CI 0.76–0.90) for ABSI score (Fig. 3). Age and TBSA% had the greatest impact on AKI development and mortality. Ten-unit increase in age and TBSA% increased the likelihood of death with an OR of 1.9 and 2.4, respectively. However, the number of deaths and AKIs was too small for a reliable multivariate analysis that included variables other than age and TBSA% (Table 2). When the adjusted model also included AKI, a ten-unit increase in age and TBSA% decreased the OR for death to 1.6 and 2.4, respectively. Moreover, among AKI patients, a ten-unit increase in TBSA% elevated the risk of death to OR 1.5. AKI increased the risk of death most as individual factor, however, as age and TBSA% being continuous variables, their impact increased in patients with extensive burns and high age.

**Fig. 4:**
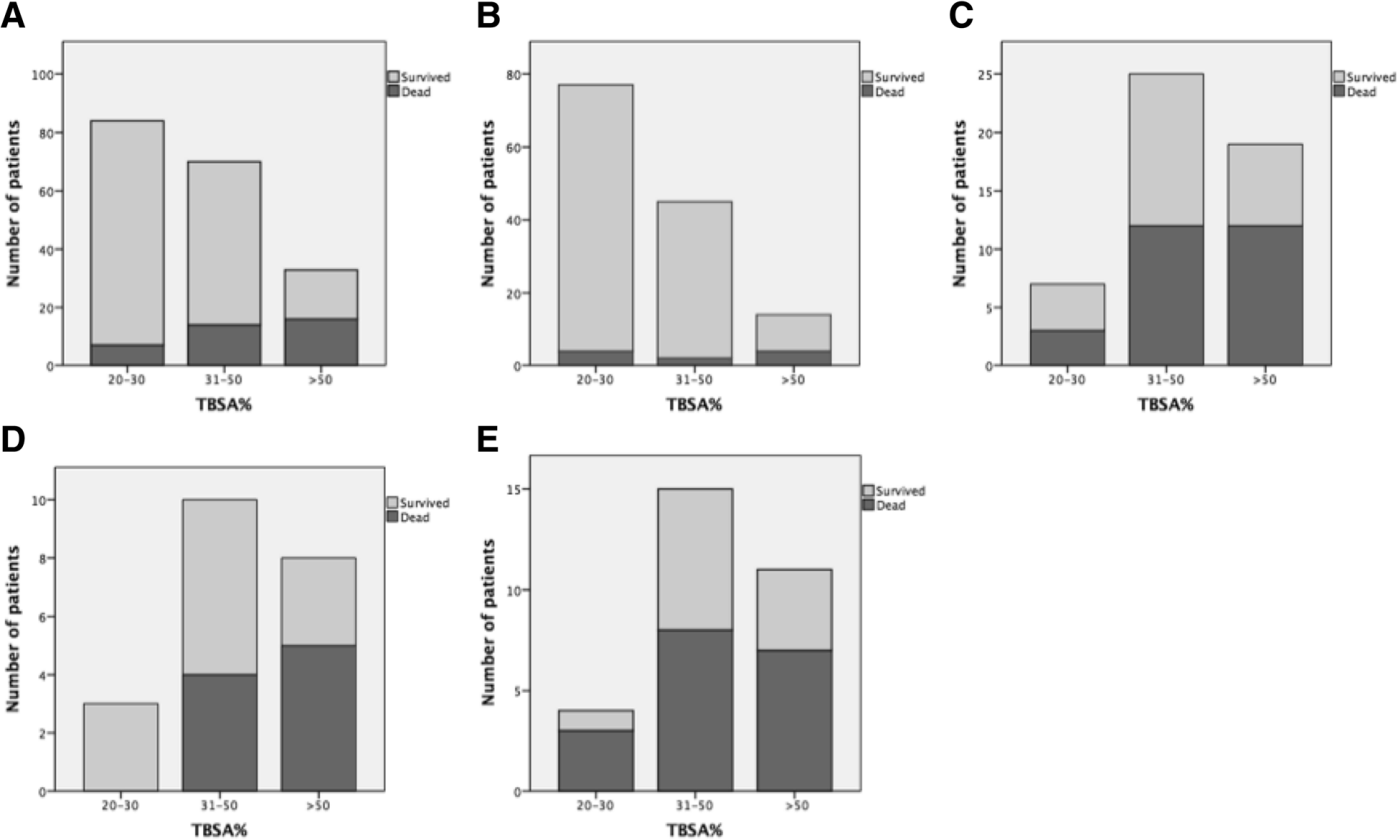
Distribution of survivors and non-survivors in **a**) all patients **b**) no AKI patients **c**) AKI patients **d**) RRT patients **e**) AKI, no RRT patients. TBSA; Total body surface area

The probability of death formed an S-shaped curve when plotted with Baux score. The probability of death rose rapidly after Baux score 80, especially in flame burns. Among all ICU patients, a Baux score of 100 predicted an approximate 40% probability of AKI with an approximate 30% probability of death. At Baux score 70 in patients with AKI, the probability of death was approximately 30%, whereas in all patients with the same Baux score the probability was approximately 5%. Among AKI patients, a linear association between probability of death and Baux score emerged. When both Baux curves predicting death or AKI were adjusted for all variables presented in Table 2, the results stayed the same (Fig. 2). A comparison of survivors vs. non-survivors in various subgroups is shown in Fig. 4.

## Discussion

### Incidence of AKI, RRT and mortality

This retrospective study investigated a cohort of 187 ICU patients (with ≥20% TBSA%) treated in the Helsinki Burn Centre from 2006 to 2015. We observed a 27.3% incidence of AKI and 19.8% overall mortality. A pooled study of 3941 burn patients from 18 non-heterogeneous studies conducted between 2007 to 2016 revealed a 39.4% incidence of AKI defined by the RIFLE criteria and 13.2% overall mortality [1]*.* Another American retrospective cohort of 1476 patients with > 20% TBSA% revealed a 20.7% incidence of AKI [18]*.* Comparisons with other studies is not straightforward due varying AKI definitions and inclusion criteria between studies; over 20 AKI definitions have been presented in past decades [2]*.* Our AKI definition of SCr > 120 μmol/l (1.4 mg/dl) did not take into account decrease in diuresis, which likely underestimates the true amount of AKI cases. We believe, that our criteria includes most of the considerable AKI cases, however, some mild AKIs are potentially not included. We also emphasize that considerable AKI is possible with SCr < 120 μmol/l (1.4 mg/dl). If we used the AKIN classification without diuresis criterion [19], 10% of AKI patients would have been considered as not having AKI and 47% to AKIN grade I because the classification is based on increase in SCr within 48 h. On the other hand, 37% of AKI patients showed SCr values > 100 μmol/l (1.1 mg/dl) and 12% had at least 150 μmol/l (1.7 mg/dl) on arrival. Accordingly, it is worth noting that among burn populations, SCr on arrival is rarely the baseline for SCr due to dehydration or already reduced kidney function; this must be taken into account as a limitation for a percentage-based increase in SCr criterion. When using a certain value of SCr as AKI definition, it is known that creatinine poorly reflects rapidly impaired renal function in fast progressive early AKI [20]*.* Moreover, as this study investigated burn patients, the results cannot be directly applied to general ICU patients due to different pathogenesis.

### Risk factors for AKI and death

Multivariate logistic regression showed that age, TBSA%, rhabdomyolysis, and sepsis were individual risk factors for AKI. However, the 95% CIs for all variables except for age and TBSA% were very wide and thus our results merely suggest their role as risk factors for AKI. The ORs for age, TBSA%, and AKI are similar to those from recent studies on severely burned patients [5, 21]*.* After excluding patients aged < 16 years, the results in multivariable analysis did not change notably.

Age, TBSA%, and AKI were significant independent risk factors for death during hospital stay. However, the number of the deaths was too small for reliable multivariate analysis for other variables (Table 2). AKI was, however, a clear risk factor for death despite the wide confidence interval. When the adjusted model also included AKI, the association between age, TBSA%, and death decreased, suggesting that AKI is part of causal pathway to death. Furthermore, age and TBSA% also increased the risk of death in AKI patients.

Inhalation injury has been confirmed in a recent pooled study as a clear risk factor for AKI [1]*.* Inhalation injury did not achieve significance in this study, possibly due to limited power. Pre-existing comorbidity had no effect on AKI or death in any analyses. In an American multicentre study of 31,338 patients, comorbidities were risk factors for poor outcome, though the impact varied depending on the type of illness. They reported that 26.4% of patients had at least one pre-existing comorbidity, whereas our value was 36.9% [22]*.* A Swedish study of 772 burn patients concluded that previous health weighted by Charlton’s index did not increase the risk of death in multivariable analysis [23]*.* As a limitation, our study did not take into account the different impact or severity of comorbidities, which may affect the outcome and lead to lack of association. Palmieri et al. also noticed that comorbidities were not more common in the AKI vs. no AKI group in 26 severely burned patients [4]*.*

Rhabdomyolysis occurred in nearly half of the electrical injuries and was strongly associated with need for RRT (*P* < 0.05). Rhabdomyolysis required significantly more often RRT when compared with the remaining AKI patients (33.3% vs. 3.2%). Rhabdomyolysis has been previously shown as a risk factor for AKI in 525 burn patients [24]*.* Rhabdomyolysis also occurred in 25% of burns caused by hot air in sauna. This association of hot-air induced rhabdomyolysis has been described earlier in Finnish studies of sauna burns [25, 26]*.*

All AKIs caused by sauna and nearly 90% of rhabdomyolysis occurred early; all of these patients survived. In unadjusted analysis, flame burns were more common in non-survivors vs. survivors (*P* = 0.03). In earlier studies, flame burns were over-represented in non-survivors due to extensive burn area, possible simultaneous inhalation injury, and depth of burn injury [3, 27]*.*

There was no difference in outcome between genders. Results from single studies are contradictory [28, 29]*.* A 16-year Swedish register study on 1119 patients did not find gender as an individual risk for death [30]*.* A 7-year American study of 1611 patients concluded that females aged < 60 years had an individual risk for death, whereas older women had no such risk [29]*.* In a recent cohort of severely burned patients, female gender was independently associated with poorer outcome [21]*.* In our sample, however, the number of females included was small.

### Outcome

This study concluded that late AKI has a poorer outcome than early AKI, which is supported by previous studies [12, 13]*.* Early AKI is mainly associated with hypovolemia and rapid impairment of glomerular filtration rate that leads to anuria, whereas late AKI is often associated with sepsis and multiple organ failure (MOF) [6]*.* The vast majority of non-survivors in the late AKI group developed MOF and isolated renal failure was rare among non-survivors. All late AKI patients that received RRT (*n* = 3) died; these patients had severe injuries (mean Baux 116, ABSI 11.3). The prognosis of different AKI types seems inconsistent, at least partially due to the fact that different definitions for onset of AKI and even for AKI itself exist [3, 6, 8, 9, 11]*.* Studies have defined AKI via SCr or diuresis alone, or by both. AKI definition, onset of AKI, and exclusion of patients with no chance of survival (death within days after arrival) are essential when assessing outcome.

Late AKI patients had significantly higher ABSI scores and TBSA% compared with early AKI patients. However, there were no differences in age or Baux score. A total of 50% of early AKI patients received RRT compared with 23.5% of late AKI patients (*P* = 0.07) (Additional file 1: Table S1). In this study, no strong evidence emerged of a better prognosis in RRT patients compared with AKI patients that did not receive RRT. One confounding factor is that 37% of AKI patients that did not receive RRT were excluded from RRT due to various factors, frailty in general, or increased amount of comorbidities (AKI without RRT 50% vs. RRT 33%).

We believe that the better prognosis of the early AKI group can be explained by lower ABSI scores and at least partly because of the early decision to initiate RRT for the patients most likely to benefit from it. However, despite the 10-year study period, the number of RRT patients was small and consequently limits our ability to make strong conclusions.

Figure 4a, b, c, d and e show that AKI has a notable impact on mortality. The mortality in major burns (TBSA > 50%) is moderate without AKI, although approximately 60% of patients in this group developed AKI during hospital stay. The incidence of AKI is relatively small (< 10%) in minor burns (TBSA ≤30%) (Fig. 4b and c). However, AKI notably increases the risk of death when observed (Fig. 4c). In the RRT group (Fig. 4d), patients with TBSA% < 50 seem to have reduced mortality compared with AKI patients that did not receive RRT (Fig. 4e). This trend line is comparable to an earlier study from the our institution, although the prognosis of RRT patients seems to have improved [3]*.*

### ABSI, Baux, and modified Baux scores as predictors for outcome

The risk of death and AKI were small with Baux scores < 80. In AKI patients, however, the risk of death rose linearly and Baux LD_50_ in AKI patients was 96 compared to a Baux LD_50_ of 112 in all patients. The risk of death in AKI patients was already seen with low Baux scores (Baux < 80). These results highlight AKI as a strong independent predictor of mortality and this is supported by previous evidence [4, 5, 21, 28]*.*

ABSI, Baux, and modified Baux scores showed nearly equal and good AUC in predicting death during hospital stay. Surprisingly, the modified Baux score was not superior to the original Baux score. The modified Baux score showed an AUC of 0.84 to 0.96 in predicting death during hospital stay in several previous studies on burn patients treated in 1987 to 2013 [31–35]*.* On the other hand, the original Baux score has proved to be very reliable, showing an AUC of 0.90 to 0.93 AUC in burn patients treated in 1977 to 1996 [36] and 2003 to 2009 [35]*.* Likewise, a Swedish study with 1946 patients conducted in 1993 to 2015 revealed an AUC of 0.97 [37]*.* We emphasize that inclusion criteria (age and TBSA%), sample size, and also exclusion criteria (death during first days from admission) have an essential effect on prediction power when comparing results between studies.

The worsening of the prediction power and increase in LD_50_ can also be explained by improved burn care. The general prognosis of burn patients has improved over time and even more patients with extensive injuries will survive [38]*.* We observed an LD_50_ for a Baux score of 112, which is improved when compared with studies based on patients treated in 1977 to 1996 (LD_50_ approximately 100) [36, 39] and is comparable to recent studies with patients treated in 2000 to 2008 (LD_50_ approximately 110) [40] and 1993 to 2012 (LD_50_ approximately 110) [41]*.* When the Baux score was presented in the early 1960s, the LD_50_ was observed at a score of 75 [15]*.* As seen in Fig. 2, the Baux score predicting mortality forms an S-shaped curve, which is comparable to a previous study with 333 adult burn patients [35]*.* Especially in flame burns, the risk of death increases rapidly when the Baux score reaches 80, which accounted for 75% of cases in the present study.

### Comparison to the earlier Helsinki Burn Centre study [3]

The average TBSA% of all patients in this study was higher (37.3% vs. 31.4%) than in a previous report published from AKI patients in our institution between 1988 to 2001. However, the mortality of all patients was slightly lower (19.8% vs. 21.4%). We highlight that in minor burns severe AKI also is possible and increases mortality. However, these patients were not included in the present study (in contrast to the previous study). The overall mortality would have been even lower if these patients were included. In the previous study, where all ICU patients regardless of TBSA% were included and SCr > 120 μmol/l (1.4 mg/dl) was the criterion for AKI, AKI patients had 44.1% mortality and 40.2% mean TBSA% (in this study we observed 52.9% mortality and 48.1% mean TBSA%). When all patients between 2006 and 2015 who received RRT regardless of TBSA% (as in the previous study) were included, we found four patients who needed RRT with TBSA% < 20 (not included into the study). By including these patients into the present study, the mortality of RRT patients would have decreased from 52.9 to 40%. Accordingly, severe AKI is possible even in minor burns. This has been reported in detail regarding the association between hot-air sauna burns and AKI [26]*.* In this study, three-fourths of AKIs due to hot-air sauna burns required RRT.

The number of RRT patients has remained at a low level (2.5 patients per year) and is nearly equal to the results from 1988 to 2001 (approximately 2.4 patients per year). While the mean TBSA% in RRT patients was slightly higher (50% vs. 43%), mortality decreased from 63 to 43%. The incidence of AKI has not changed substantially but the proportion of RRT among AKI patients increased from 26.1 to 53.6%. The increased availability of RRT and improved ICU treatment can explain these positive outcomes. A systematic Belgian review revealed an overall decreasing trend of mortality in AKI and RRT patients in burns during the years 1960 to 2009 [2]*.*

### Strengths and limitations

Our study has some strengths. Patient selection and AKI definition were clear and the size of the study population provided sufficient statistical power for most of the factors under investigation. However, this was a retrospective, single-centre study and some aspects remained unclear due to small subgroup size. The AKI definition likely underestimated the true amount of AKI cases. We emphasize that the definition of AKI differs in similar studies and poses challenges when interpreting the results between studies or pooling data. Similarly, the definition of sepsis in burn patients is often unclear since all patients sustaining severe burn have SIRS (systemic inflammatory response syndrome) and often times one cannot distinguish it from sepsis. Also, RRT was withheld from some AKI patients, which biases potentially the outcome. Since this cohort included only burn patients, the results are not directly applicable to general ICU patients.

## Conclusions

This study demonstrated that age, TBSA%, and AKI are the strongest independent factors that predict outcome in severe burns. Early AKI patients seemed to have a better prognosis compared with late AKI patients. Even a major burn injury without AKI had a relatively good prognosis. However, the likelihood of AKI increases with increasing Baux score. The Baux score was related to the probability of AKI and death and showed an S-shaped curve; the risk of these two outcomes increased rapidly after a Baux score of 80. In addition, the probability of death rose linearly with increasing Baux score in AKI patients. Protective evidence for RRT in preventing death was not confirmed. However, biased patient selection and small sample size were notable limitations. The prognosis of burn injury has improved over time, but even small increases in SCr during ICU stay notably increase the risk of death in severely burned patients. Interventions to prevent AKI are necessary to improve the prognosis of patients with severe burns.

## Additional file

Additional file 1: **Tables S1.** Demographic data of early and late AKI patients. (DOCX 15 kb)

## Abbreviations

ABSI: The Abbreviated Burn Severity Index
AKI: Acute kidney injury
CRRT: Continuous renal replacement therapy
ICU: Intensive care unit
IHD: Intermittent hemodialysis
LD_50_: 50% lethal dose (50% likelihood of death)
P-CK: Plasma creatinine kinase
RRT: Renal replacement therapy
SCr: Serum creatinine
