# Supplementary material for: Factors associated with acute kidney injury in the Helsinki Burn Centre in 2006–2015
Source: Scand J Trauma Resusc Emerg Med. 2018 Dec 13;26:105. doi: 10.1186/s13049-018-0573-3 (PMC6293617; doi:10.1186/s13049-018-0573-3)
Supplement: Supplementary file 1 — Tables S1. Demographic data of early and late AKI patients. (DOCX 15 kb) [file 13049_2018_573_MOESM1_ESM.docx]

**Additional file 1: Table S1.** Demographic data of early and late AKI patients

| Variable | Early AKI (n=34) | Late AKI (n=17) | P |
| --- | --- | --- | --- |
| Age | 54.1 ± 17.4 (17-87) | 50.6 ± 20.0 (19-83) | 0.52 |
| Burned TBSA | 43.7 ± 16.5 (20-80) | 56.9 ± 15.3 (31-84) | **<0.0001^*^** |
| Burn mechanism  Flame  Sauna (hot air)  Electrical  Liquid  Explosion  Steam | 28 (82.4 %)  4 (11.8 %)  0 (0 %)  1 (2.9 %)  1 (2.9 %)  0 (0 %) | 15 (88.2 %)  0 (0 %)  1 (5.9 %)  1 (5.9 %)  0 (0 %)  0 (0 %) | 0.70 |
| ICU stay time (days) | 29.6 ± 22.9 (3-90) | 34.9 ± 21.1 (9-85) | 0.43 |
| ABSI-score | 9.5 ± 1.5 (6-12) | 10.6 ± 1.6 (7-13) | **0.02^*^** |
| Baux score | 97.9 ± 16.3 (59-124) | 107.5 ± 19.8 (64-141) | 0.07 |
| Male sex | 23 (67.6 %) | 14 (82.4 %) | 0.33 |
| Inhalation injury | 9 (26.5 %) | 5 (29.4 %) | 1.00 |
| Intubated on arrival | 27 (79.4 %) | 11 (64.7 %) | 0.32 |
| Escharotomies/fasciotomies | 30 (88.2 %) | 15 (88.2 %) | 1.00 |
| Pre-existing co-morbidity | 15 (44.1 %) | 7 (41.2 %) | 0.84 |
| Sepsis | 6 (17.6 %) | 6 (35.3 %) | 0.18 |
| Rhabdomyolysis | 7 (20.6 %) | 1 (5.9 %) | 0.24 |
| RRT | 17 (50.0 %) | 4 (23.5 %) | 0.07 |
| Non-survivors | 16 (47.1 %) | 11 (64.7 %) | 0.23 |

*) Statistically significant difference, p < 0.05

Data are reported as mean ± SD, (interval) or percentage, when appropriate. ABSI, Abbreviated Burn Severity Index; AKI, acute kidney injury; ICU, intensive care unit; RRT, renal replacement therapy; TBSA, total body surface area.
